# A phase 1 clinical trial of a multi-antigen SARS-CoV DNA vaccine as a booster after dose three spike-based mRNA vaccinations

**DOI:** 10.1016/j.omta.2026.201710

**Published:** 2026-02-28

**Authors:** Soo Aleman, Gustaf Ahlén, Jingyi Yan, Per Ljungman, Peter Bergman, Christian An Binh Nordentoft, Sofia Appelberg, Arlisa Alisjahbana, Matteo Cadossi, Simona Salati, Katja Tobin, Ola Tuvesson, Hanna Tegel, Eva-Karin Gidlund, Friedemann Weber, Olivia Larsson, Urban Höglund, Marcus Buggert, Sophia Hober, Lars Frelin, Ali Mirazimi, Matti Sällberg

**Affiliations:** 1 Department of Infectious Diseases, Karolinska University Hospital, Stockholm, Sweden; 2 Department of Medicine Huddinge, Karolinska Institutet, Stockholm, Sweden; 3 Department of Laboratory Medicine, Karolinska Institutet, Stockholm, Sweden; 4 Clinical Microbiology, Karolinska University Hospital, Stockholm, Sweden; 5 Department of Cellular Therapy and Allogeneic Stem Cell Transplantation, Karolinska University Hospital, Karolinska Comprehensive Cancer Center, Stockholm, Sweden; 6 Public Health Agency of Sweden, Stockholm, Sweden; 7 Center for Infectious Medicine, Department of Medicine Huddinge, Karolinska Institutet, Stockholm, Sweden; 8 IGEA Spa, Carpi, Italy; 9 Department of Clinical Cancer Studies, Karolinska University Hospital, Stockholm, Sweden; 10 Northx Biologics, Matfors, Sweden; 11 Institute for Virology, FB10-Veterinary Medicine, Justus-Liebig University Giessen, Giessen, Germany; 12 Adlego Biomedical AB, Uppsala, Sweden; 13 Department of Protein Science, KTH – Royal Institute of Technology, Stockholm, Sweden; 14 Karolinska ATMP Center, Stockholm, Sweden; 15 Department of Clinical Immunology and Transfusion Medicine, Karolinska University Hospital, Stockholm, Sweden; 16 Medical Diagnostics Karolinska, Karolinska University Hospital, Stockholm, Sweden

**Keywords:** DNA vaccine, clinical trial, COVID-19

## Abstract

The COVID-19 pandemic was controlled by active vaccination, but emergence of viral mutations in the receptor-binding domain (RBD) of the spike (S) protein supported the need for vaccines targeting genetically stable viral domains. We developed a unique vaccine (OC-007) combining WH1-, Alpha-, and Beta-variant RBD loops; the membrane (M; WH1); and nucleoproteins (N; WH1). In a first-in-human, placebo-controlled, randomized, double-blind, dose-escalation phase 1 clinical trial, the OC-007 DNA vaccine was evaluated as one booster dose delivered intramuscularly by *in vivo* electroporation (EP) in 16 healthy volunteers (HVs) who had received three mRNA vaccinations. The HVs received 0.5, 1.0, or 2.0 mg of OC-007 or placebo (NaCl) by EP and were followed for 3 months. The OC-007 vaccine was safe and tolerable with no severe adverse events. There was a 2.0- to 5.7-fold increase in antibody levels to the S Beta variant and to the N protein in the 2.0 mg dose group, in which two participants had a COVID-19 diagnosis on days 21 and 28, respectively. Neutralizing antibody levels increased to SARS-CoV-2 Beta (1.0 and 2.0 mg groups) and Omicron XBB1.5 variants (2.0 mg group). The novel OC-007 DNA vaccine was safe, tolerable, and induced/boosted T cell responses to genetically stable proteins.

## Graphical abstract

**Figure:**
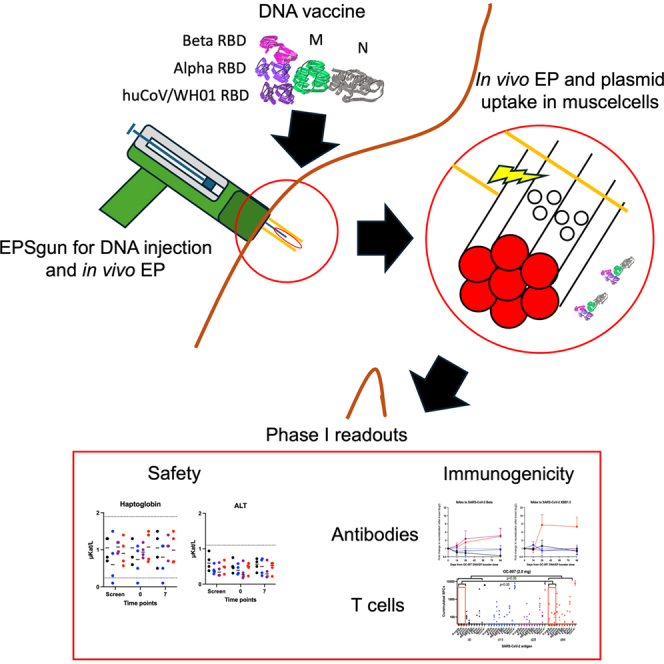

## Introduction

The impact of the COVID-19 pandemic on healthcare and society was reduced by the global use of vaccines targeting the spike (S) protein or inactivated whole virus (INA) vaccines.^1^^,^^2^^,^^3^^,^^4^ However, during the pandemic, SARS-CoV-2 evolved from the relatively infectious wild-type virus (WH-1) to the highly infectious and mutated Omicron variants (BA.1-5, XBB, JN-1, etc.^5^). Thus, the virus adapted to the human host through the accumulation of mutations. These mutations were frequently located to the receptor-binding domain (RBD) and mediate escape from neutralizing antibodies, or to non-structural regions to improve the replicative capacity.^5^^,^^6^^,^^7^ Hence, the next-generation of vaccines should target more genetically stable regions of the virus to ensure broader cross-reactivity.^8^ Recent data from 2023 to 2024, show that the vast majority (88%) of adults hospitalized due to COVID-19 had not received the updated vaccines.^9^ This reinforces the importance of repeated vaccination with updated vaccines in the older age groups or the development of vaccines targeting more conserved regions of the virus. All approved genetic or protein-based COVID-19 vaccines contain only the spike protein, whereas INA vaccines may contain all structural proteins and multiple strains of SARS-CoV-2. Genetic vaccines, such as mRNA, various adenoviral vectors, and DNA, have the ability to induce both antibody and CD4+ and CD8+ T cell responses. In contrast, protein-based and INA vaccines mainly induce antibody and CD4+ T cell responses. There is strong evidence that T cells, both CD4+ and CD8+, are essential for providing long-term protection against severe COVID-19 and mortality.^10^ Thus, it is necessary that these T cells target genetically stable regions of the virus to minimize the risk of mutations accumulating in T cell epitopes. However, considering the outbred nature of the human leukocyte antigen system, this is much less likely to occur in viruses only causing acute infections as compared to those causing persistent infections. Nonetheless, as many RNA viruses with a zoonotic origin have the ability to jump between animal species and replace large domains of their genome by recombination, vaccines that contain multiple viral proteins offer a long-term advantage, as they induce broader T cell responses to all components of the vaccine.

Based on the above, we developed a new chimeric vaccine gene, termed OC-007, that contains three RBD loops from the WH1, Alpha, and Beta variants of SARS-CoV-2, and the genetically stable membrane (M) and nucleocapsid (N) proteins of the WH1 variant.^11^ We have shown that when this vaccine gene is delivered as DNA by *in vivo* electroporation (EP), it effectively induces high levels of S-specific antibodies, neutralizing antibodies, and T cell responses in animal models.^12^ Importantly, the vaccine mediates protection against lethal disease in the human ACE-2 transgenic mouse model.^11^ Also, safety studies in rabbits demonstrated that the vaccine was safe and tolerable.^11^

We aimed to evaluate the OC-007 DNA vaccine delivered intramuscularly by *in vivo* EP as a fourth booster dose following three prior doses of mRNA vaccines in a first-in-human study involving healthy volunteers. Overall, we found that the vaccine was safe, well tolerated, and immunogenic, capable of priming and boosting T cell responses to the M and N proteins that none of the approved genetic vaccines can induce.

## Results

### Participants

Twenty-four healthy volunteers were screened after providing informed written consent. Eight were excluded due to screening failures including abnormal biochemical tests (hemoglobin, thrombocytes, D-dimer, creatinine phosphokinase-MB [CPK], creatinine), a previous upper-body operation with a metal plate, drop-out after screening, and body mass index (BMI) below the inclusion criteria.

Sixteen study participants were included in the study, with a mean ± SD age of 37 ± 13 years (range 19–56) (Table S1). Ten were men (62.5%), and six (27.5%) were women. The mean BMI ± SD was 25.2 ± 2.8 kg/m^2^. They all had received three doses of registered mRNA COVID-19 vaccines Comirnaty (Pfizer), Spikevax (Moderna), or their combination, with the latest dose received ≥3 months prior to the screening. The mean ± SD time between the last received mRNA vaccine dose and the administered study vaccine was 16 ± 5 months.

### Safety and tolerability

The causality of all adverse events (AEs) in the study was determined by the principal investigator, according to prespecified definitions in the study protocol approved by the European Medical Agency. There was no serious adverse event (SAE) observed in the study. The reported AEs were mainly due to pain at the time of the vaccination including the EP, local, and systemic reactogenicity (Table 1).

#### Pain at vaccination

**Figure 1:**
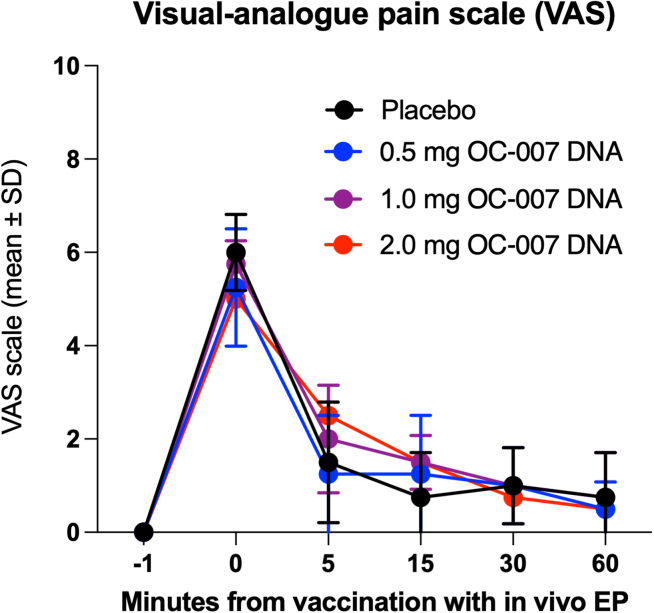
The pain level during and after *in vivo* EP was recorded using the VAS Data have been given as group means at each time point.

All 16 subjects reported pain at the time of the injection (Figure 1). The median intensity of discomfort according to the visual analog scale (VAS) was 6 (min-max, 2-7). After 5 minutes, 14 of 16 still experienced some pain (median 2; min-max, 0-4). During the 1-hour follow-up, the number of patients still experiencing pain decreased (12/16 at 15 min, 11/16 at 30 min, and 9/16 at 60 min post-vaccination). The intensity also decreased over time, with a median VAS of 1.5 (min-max, 0-3) at 15 min, a median of 1 (min-max, 0-2) at 30 min, and a median of 1 (min-max, 0-2) at 60 min among those participants still experiencing pain. There were no significant differences in pain intensity at any time point between participants receiving the different dose levels or placebo (data not shown).

#### Other AEs

Reported AEs, other than the injection site pain and their severity, are shown in Table 1. Overall, 41 other AEs were reported in 15 of the 16 participants. One was assessed as being of severe intensity (CPK increase at day 7 post-vaccination) and was judged to be possibly or likely related to the study procedure (i.e., related to study vaccine or placebo/EP) by the responsible investigator. This participant had, however, performed excessive exercise days prior to the blood sampling as another potential explanation for the increase of the CPK level. The CPK level returned spontaneously to baseline in a subsequent sample. Six AEs were moderate (all in patients receiving the study vaccine), of which four were regarded possibly or likely related to the study procedure. The remaining 34 AEs were graded as mild, of which 17 were regarded as possibly or likely related to the study procedure.

Nine AEs occurred (other than the pain directly at the time of the injection) in the low-dose group: six mild, two moderate, and one severe. Three of these were regarded unrelated to the study procedure. In the mid-dose group, nine AEs were reported: eight mild and one moderate, of which five were regarded as unrelated to the study procedure. In the high-dose group, 11 AEs were reported: eight mild and three moderate, of which four were considered as unrelated to the study procedure. In the placebo group, 12 AEs were reported: all mild, of which five were regarded as possibly or likely related and seven as unrelated to the study procedure.

#### Laboratory abnormalities

Four participants showed laboratory abnormalities 7 days post-vaccination (Figures S1 and S2). One participant had increased levels of serum aspartate aminotransferase, lactate dehydrogenase, and CPK, which were judged as being possibly or likely related to the vaccination. Two other participants had increased serum creatinine levels and one had a mild decrease in hemoglobin; all three were in the placebo group and judged as unrelated to the study procedures.

### COVID-19

Three participants tested positive for SARS-CoV-2 during the study. One participant who was asymptomatic and tested negative on SARS-CoV-2 antigen test was found to be SARS-CoV-2 positive at the PCR test at day 1, prior to receiving the study drug vaccine. Two other participants had antigen/PCR-verified SARS-CoV-2 infection on days 30 and 33, respectively, after receiving the study drug vaccination. All three participants experienced mild, grade 1 COVID-19,^13^ with no requirement for hospitalization.

### Antibody responses

The antibody responses were analyzed by examining individual values, changes from baseline, and geometrical means. The sample size was too small for reliable statistical comparisons, whereby results are mainly presented as descriptive statistics and grouped comparisons. All participants were positive for one or more SARS-CoV-2 antigens already at inclusion, and they had variable antibody levels prior to the booster vaccination.

**Figure 2:**
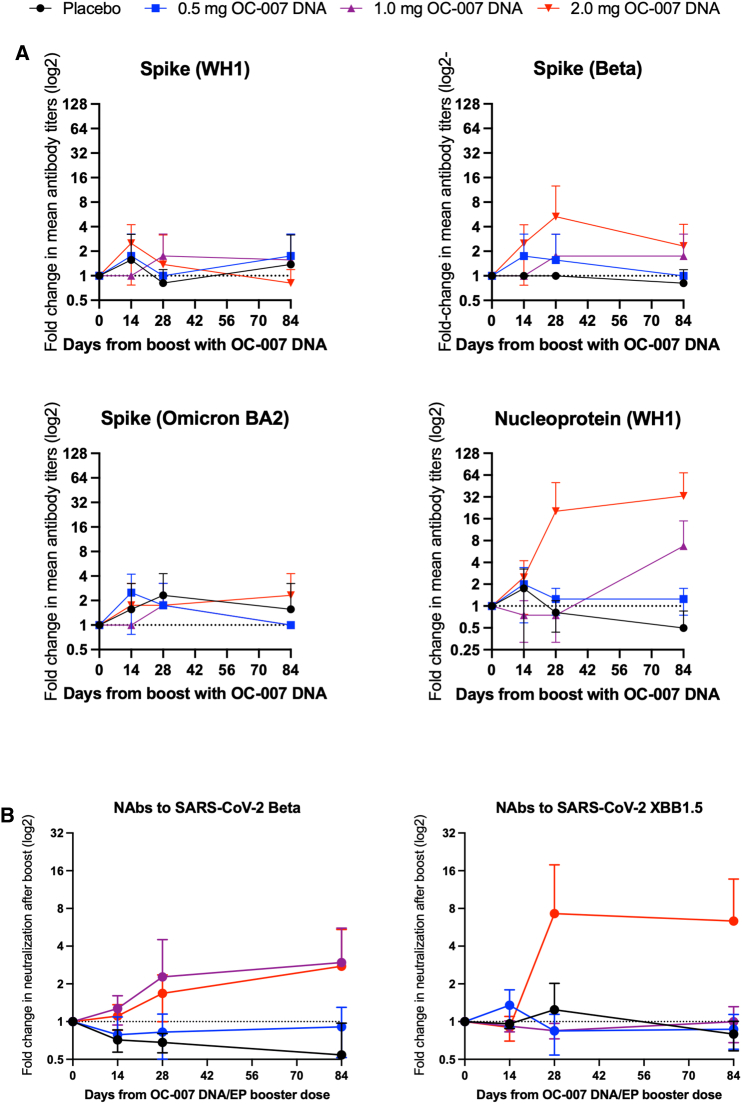
Analysis of humoral immune responses to SARS-CoV-2 Changes in the levels of antibody responses in EIA (A) to the Spike proteins corresponding to the WH-1, Beta, and BA2 Omicron variants, and to the nucleoprotein of the WH-1 variant. Also shown in the change in levels of neutralizing antibodies (NAbs) to the Beta and the Omicron XBB1.5 variants using live virus (B). Data have been given as the log2 change (+ standard deviation) in antibody levels as compared to before the booster dose with the OC-007 DNA vaccine.

**Figure 3:**
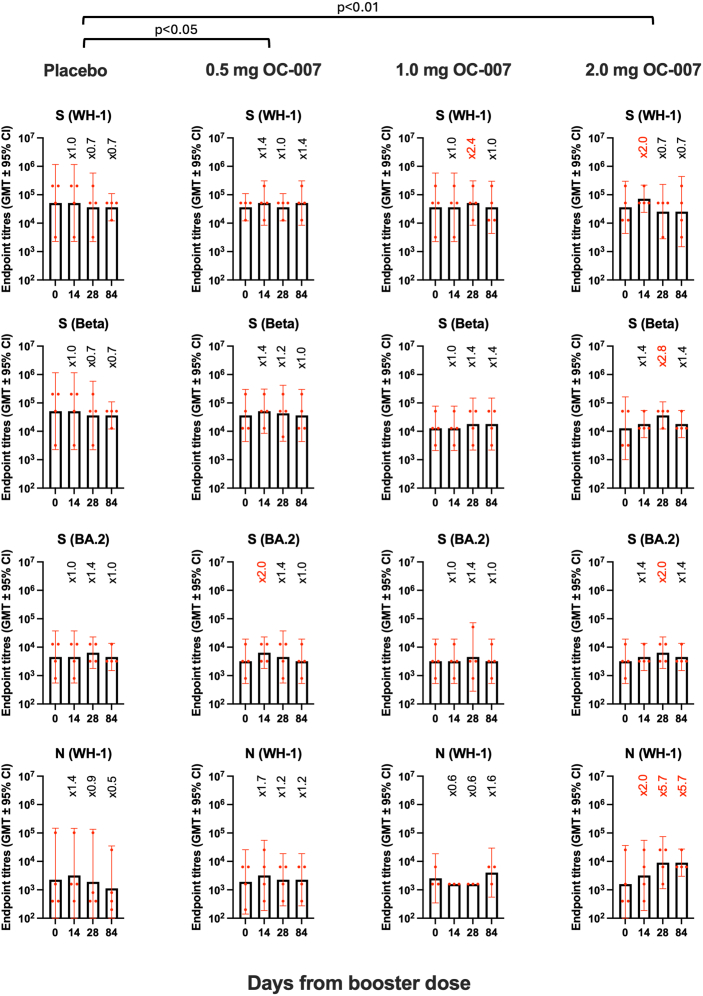
Mean geometric mean antibody endpoint titers (GMT ± 95% CI) to spike proteins of the WH-1, Beta, and BA.2 variants, and the WH-1 nucleoprotein by EIA The data from each group and each antigen have been given separately. The groups were compared statistically using the fold change in GMT using the Mann-Whitney U test. CI, confidence interval.

The antibody levels to the spike proteins corresponding to the WH1, Beta, Omicron BA.2, and the N protein (WH1) were determined by an in-house developed enzyme-linked immunosorbent assay (ELISA). The mean ± SD fold change in geometric mean titers (GMT) before and at all time points after the booster was compared using four different antibody assays (Figure 2). The mean GMT change after vaccination in the placebo group was 0.9 ± 0.3 vs. 1.3 ± 0.3 in the 0.5 mg group (*p* < 0.05 for 0.5 mg vs. placebo; Mann-Whitney U test; Figure 3), vs. 1.2 ± 0.5 in the 1.0 mg group (*p* > 0.05 for 1.0 mg vs. placebo; Mann-Whitney U test; Figure 3), and vs. 2.3 ± 1.7 in the 2.0 mg group (*p* < 0.01 for 2.0 mg vs. placebo; Mann-Whitney U test; Figure 3). Overall, an increase in GMT (>1.0) from baseline at any time point after vaccination with OC-007 was more common in the vaccine groups compared to the placebo group (2/12 vs. 24/36; *p* < 0.01, Fisher’s exact test). The most pronounced increases in antibody titers were seen in the 2.0 mg group, in which two participants were positive for SARS-CoV-2 on days 30 and 33, respectively. Thus, these infections most likely influenced the results from days 28 and 84. However, 2-fold increase in GMTs were observed against S (WH-1) and N (WH-1) already by day 14, suggesting that the strong responses seen on day 28 may reflect a combination of vaccine-induced and recall responses in these participants.

Increases in titers achieving 50% neutralization (NT_50_) from baseline to the live SARS-CoV-2 Beta variant were seen in the 1.0 mg (3-fold) and the 2.0 mg (2.7-fold) groups (Figure 2B). Interestingly, the NT_50_ to the XBB variant was only seen in the 2.0 mg group and on days 28–84. This would suggest that the Beta variant-specific NT increases were vaccine induced, whereas the XBB responses were related to both the infections and the vaccinations, as these only appeared in the 2.0 mg group. The individual changes in NT titers have been given in Figure S3.

Overall, considering the limited size of the study, the antibody responses to the OC-007 DNA vaccine appear to be dose-dependent, with the most pronounced responses observed in the 2.0 mg group. When analyzing the antibody data without the three subjects who had COVID-19 infections during the study period it was shown that most statistical comparisons held up, but the changes were of a lesser magnitude, with 2-fold increases in GMTs to the three spike and the N antigens tested. Overall, the increases tended to be somewhat lower in magnitude than those seen with BNT162b2 mRNA vaccine, where a fourth dose results in GMT increases of 4.4-fold to S of the WH-1 variant and 5.7-fold to the BA.4/5 variant in surrogate NT assays.^14^ Overall, the responses to a fourth mRNA dose were less pronounced as compared to the effects of the second and the third doses but still seem to protect against severe disease.^15^^,^^16^^,^^17^

### T cell responses

**Figure 4:**
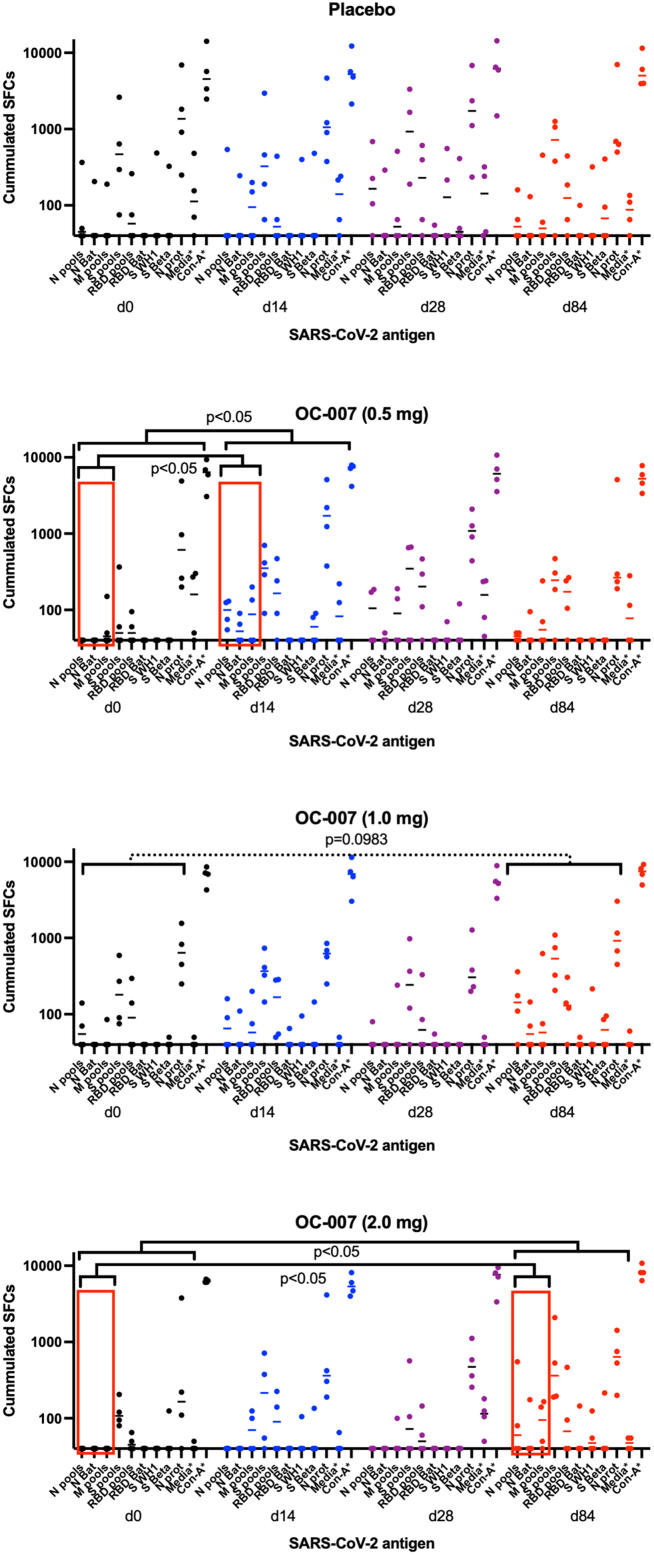
The T cell responses following vaccination with the OC-007 DNA vaccine was monitored using an IFNγ ELIspot assay (Mabtech) Data have been given as the cumulated spots to one or more peptide pools covering a SARS-CoV-2 (WH-1) antigen at days 0 (black), 14 (blue), 24 (purple), and 84 (red) after OC-007 DNA vaccination. Each dose group has been reported separately. The media control has been subtracted from all samples, except for the media and Con-A controls. Statistical comparisons were made using the Mann-Whitney U test. The data from the media and the Con-A controls have been given as the raw data from individual assays.

The T cell responses to S, RBD, M, and N were determined by interferon (IFN) γ ELISpot assay in all samples. For the statistical comparisons, only the cumulated IFNγ responses to the peptide pools (i.e., adding the spot-forming cells to the individual pools spanning the M, N, or RBD sequences) and to the individual proteins were used. By comparing baseline levels with those at days 14, 28, and 84, we noted some differences. It should be noted that this is a small study and any differences, statistical or not, should be interpreted with care. In the placebo group, there were no significant changes in the frequency of T cell responses to the different antigens with time (Figure 4; not significant (NS), Fisher’s exact test). In the 0.5 mg group, there was a significant increase in the frequency of responses 14 days after the booster dose (Figure 4; *p* < 0.05, Fisher’s exact test). In particular, the number of T cell responses to N- and M-derived antigens was significantly increased by day 14 (Figure 4; *p* < 0.05, Fisher’s exact test). This suggests that the OC-007 DNA vaccine was effective in priming, or boosting, T cell-targeting highly conserved viral proteins. In the 1.0 mg dose group there was no statistical increase in T cell responses (Figure 4; NS, Fisher’s exact test). In the 2.0 mg group, there was a significant increase in the number of responses to all peptide pools at day 84 as compared to prior to the boost vaccination (Figure 4; *p* < 0.05, Fisher’s exact test). Also here, the number of T cell responses to N-derived antigens was significantly increased by day 84 (Figure 4; *p* < 0.05, Fisher’s exact test). However, it should be noted that two subjects had mild COVID-19 infections around days 30–33, which most likely influenced the results.

Finally, the total number of positive T cell responses did not differ in the placebo group before and after vaccination (Figure 4; 16/36 vs. 56/108, NS, Fisher’s exact test). In contrast, when adding the three vaccine groups together, the total number of positive T cell responses increased significantly after vaccination (Figure 4; 34/108 vs. 148/324, *p* < 0.01, Fisher’s exact test).

**Figure 5:**
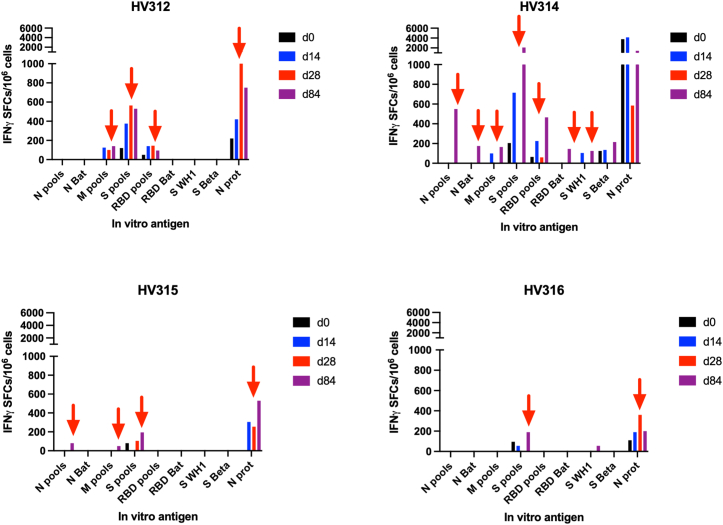
The ELISpot data from the individual subjects in the 2.0 mg dose group shown as the cumulated spot-forming cells (SFCs) per million cells The red arrow indicates a response >2 times the day 0 sample.

**Figure 6:**
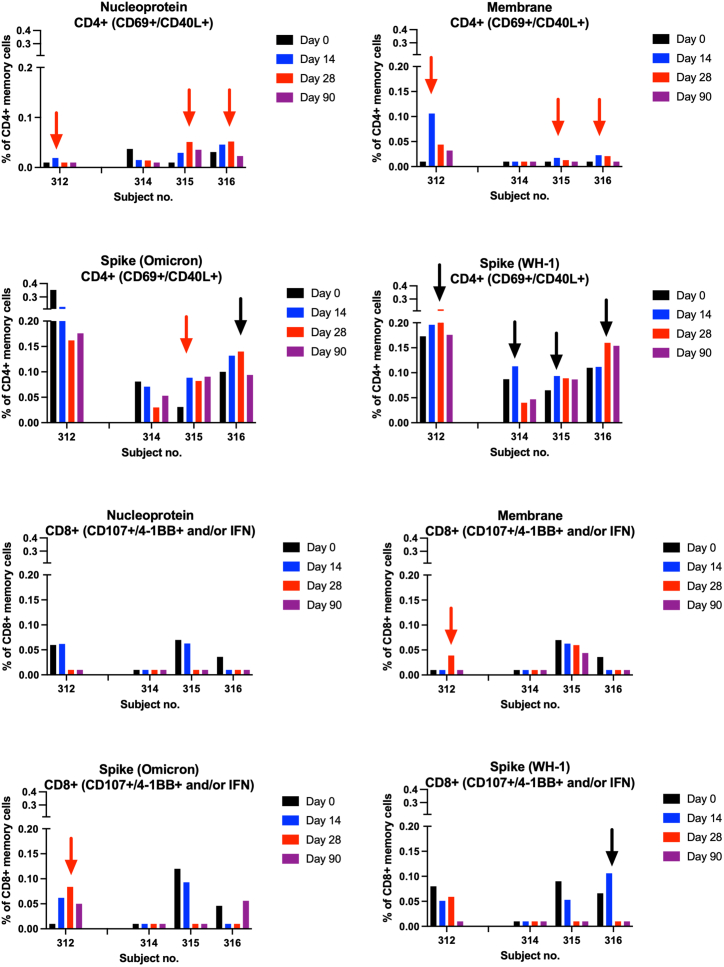
The flow cytometric analysis using activation-induced markers of the individual subjects in the 2.0 mg dose group is shown with the respective antigens used for activation The red arrow indicates a response >2 times the day 0 sample. A black arrow indicates an increase <2 times the day 0 sample.

The T cell responses were further evaluated by flow cytometry using large peptide pools to define the reactive phenotype (Figures 5, 6, and S5). The responses in the 2.0 mg group compared well between the IFNγ ELISpot data and the flow cytometric data (Figures 5 and 6). This showed that the responses in the IFNγ ELISpot often were paralleled by a CD4+ response in the flow cytometric analysis (Figures 5 and 6). In one subject with COVID-19 diagnosed around day 30 (HV312), T cells to M and S peptide pools and N protein were detected by ELISpot at days 14–84 (Figures 5 and 6). The day 14 responses most likely reflect the vaccine-primed T cells, whereas the later responses were a combination of the vaccine and the COVID-19 infection. Here, the phenotype was found to be CD4+ T cells to N and M and CD4+ and CD8+ T cells to M and spike (Omicron) pools (Figures 5 and 6). In the other (HV314) subject with COVID-19 infections, around day 30 in the 2.0 mg dose group an increase in CD4+ T cells to the spike pool was noted in the flow cytometric analysis (WH-1; Figures 5 and 6). The ELISpot data were stronger in this subject, and in particular to the spike peptide pools (Figures 5 and 6). A possible explanation for the discrepancy between the ELISpot and flow cytometric assays in this subject might be that the analyses were run on separated time points using separate tubes of frozen peripheral blood mononuclear cell (PBMC), with potentially different quality of the cells. Also, the day 0 sample had a higher reactivity in the flow cytometric analysis, potentially masking any vaccine or infection-induced activation. Finally, in the two subjects in the 2.0 mg group without COVID-19 infections, activation of T cells to the N, M, and S (Omicron) peptide pools, and N protein, were seen by ELISpot (Figures 5 and 6). The flow cytometric analysis suggested that these were mainly of the CD4+ phenotype (Figures 5 and 6). Overall, the OC-007 DNA vaccine activated mainly CD4+ T cells, and also some CD8+ T cell responses.

## Discussion

Most COVID-19 vaccines contain the spike region of SARS-CoV-2 as a gene or a protein. To date, the only approved vaccines that contain other regions of the virus are the INA vaccines, mainly used in Asia, Africa, and South America.^17^ However, as the virus continuously mutates mainly in the spike region, and in particular in the RBD domain, other viral components that represent genetically stable regions are of interest for incorporation into next-generation SARS-CoV vaccines.^7^^,^^8^^,^^18^

We, therefore, designed a new type of vaccine gene^11^^,^^12^ that was evaluated in a phase 1 first-in-human trial, with safety as the primary endpoint. We have previously shown that DNA vaccines are highly immunogenic in non-human primates and that they can protect against severe disease caused by various infections.^12^^,^^19^^,^^20^ Overall, we found that the OC-007 DNA vaccine delivered by EP was safe and tolerable, with no SAEs observed. The reported AEs were mainly related to local or systematic reactogenicity. One severe case of elevated CPK levels was observed, with a likely or possible association with the study procedure. This is most likely related to the EP or excessive training, considering the previously reported increase of CPK level after EP.^21^ The elevated CPK level resolved spontaneously, thus not indicating any serious safety concern. The electrical pulses by *in vivo* EP resulted in an immediate pain, which was quite intensive with a median VAS of 6. It is clear that *in vivo* EP constitutes a limitation for a pandemic vaccine. The need for an advanced delivery device would require vaccinations to be performed at more centralized locations, until handheld, or even disposable devices for *in vivo* EP have been developed. In addition, the pain related to the two electrical pulses may also be a limiting factor that needs to be considered, where insulation of the electrodes may alleviate some of the discomfort. Although the pain was transient with a quick decrease in intensity, it remains as a clear limitation of this vaccine approach. An advantage of *in vivo* EP for the delivery of DNA is that any DNA vaccine can immediately be combined with *in vivo* EP without any formulation. Having said that, current delivery of DNA by *in vivo* EP is this far better suited for special indications and in hospital settings for rare or chronic infections. For pandemic purposes and mass vaccination, simpler devices or alternative methods such as formulation of the DNA vaccine in lipid nanoparticles or polyplexes should be developed.

The immunogenicity of the OC-007 DNA vaccine seemed lower than the approved mRNA vaccines, although the difference between third and a fourth vaccine dose is generally less pronounced that between the second and third. The immunogenicity of the OC-007 DNA vaccine should be compared to that observed in animal models, to determine how predictive these models are. In rabbits, we analyzed the highest human dose per kg/body weight (bw) and found that this dose effectively induced both antibodies and T cells to SARS-CoV-2.^11^

It was recognized early on that a prompt T cell response during COVID-19 infection was beneficial^22^^,^^23^ and that a T cell response was related to a milder disease outcomes.^23^ Importantly, T cells recognize different parts of the virus and viral proteins as compared to B cells, making them less susceptible to the effects of mutations in the RBD domain.^24^ Recent data suggest that T cells play a pivotal role in the long-term protection against severe infection and death. In addition, the T cell responses primed by the SARS-CoV outbreak in 2003 are detectable 17 years later and are cross-reactive with proteins from SARS-CoV-2.^22^ Finally, we recently demonstrated that T cells induced by smallpox vaccinations back in the 1960s and 1970s can still be detected 40–50 years later and show cross-reactivity with the mpox virus clade II, the strain responsible for the current outbreak.^25^ Overall, substantial evidence suggests that sequences from highly conserved proteins may offer a long-term protection against severe disease. We, therefore, designed a completely new vaccine strategy, OC-007, that encompasses three RBD loops corresponding to the WH1, Alpha, and Beta variants of SARS-CoV-2, together with the M and N proteins from the WH1 variant. In animal models, we have previously shown that when delivered as a DNA vaccine using *in vivo* EP, this vaccine effectively induces broadly binding and neutralizing antibodies and highly cross-reactive T cells.^11^^,^^12^^,^^26^ In particular, T cells against the M and N proteins cross-react with homologous sequences from coronaviruses present in animals.^26^ In addition, this type of vaccine can be rapidly adapted to new SARS-CoV-2 RBD variants and still activate broadly cross-reactive T cells that recognize conserved parts of the virion. For example, M- and N-specific T cells can provide T cell helper signals to B cells that have bound and processed SARS-CoV-2 from any emerging variant. In addition, M- and N-specific cytotoxic T cells can eliminate SARS-CoV-2-infected cells corresponding to any future variant, as long as the M or N gene has not been replaced.

We, therefore, evaluated OC-007 as a booster DNA vaccine following three doses of a spike-based mRNA vaccine. The primary aim was evaluating the safety and reactogenicity of the OC-007 DNA vaccine delivered by in *vivo* EP, while the exploratory aims were to assess the vaccine-primed immune responses. A single booster dose of the OC-007 vaccine was found to be safe and tolerable, with no SAEs reported in any test group. The most frequent AE was the short-duration pain related to the *in vivo* EP. The vaccine induced increases in antibody responses mainly to the spike of Beta variant and the N protein. This was evident in the *in vitro* NT of the wild-type Beta virus in the two highest dose groups. The increase in N antibodies was most evident in the 2.0 mg dose group, but this was confounded by the fact that two of the subjects experienced mild SARS-CoV-2 infections around days 30–33. However, the mean N antibody levels had already increased 2-fold by day 14, indicating a response to the vaccination itself. Thus, the strong increases observed at days 28 and 84 are most likely a combined effect of the booster dose and the infection. As a comparison, one subject in the placebo group was diagnosed with COVID-19 on the day of vaccination. In this subject, no increase in antibody or T cell responses was observed for any of the tested antigens. However, the exact timing of the subject’s SARS-CoV-2 infection remains unknown. The levels of responses after a fourth dose determined by severity of the infection using an attenuated whole virus vaccine did seem to be weaker than from an mRNA boost, despite that the whole virus vaccine contains multiple antigens.^4^ This may reflect the inability of the whole virus vaccine to induce broader CD4+ and CD8+ T cell responses, which are induced by the mRNA vaccines and our DNA vaccine. Our vaccine also seems to induce weaker responses, but does activate CD4+ and CD8+ T cell responses to multiple antigens.

With respect to T cell responses, no significant changes were noted in the placebo group during follow-up, whereas increases were seen across all three vaccine groups collectively, as well as in the separate groups. In the 0.5 mg and the 2.0 mg dose groups, there was a significant increase in responses to the S, M, and N antigens at days 14 and 84, respectively. Importantly, there was a significant increase in T cell responses to the M and N peptide pools at the same time points. In the two highest dose groups, there was a more than 2-fold increase in T cell responses. This suggests that the OC-007 vaccine indeed induced or boosted broader T cell responses as compared to the approved mRNA vaccines, since neither M nor N proteins are present in the mRNA vaccines. A comparison between ELISpot and flow cytometric analysis suggested that the induced T cells in the 2.0 mg group were mainly CD4+ T cells, and also some CD8+ T cells.

In conclusion, the OC-007 DNA vaccine administered by *in vivo* EP was safe and tolerable and induced novel immune responses. This suggests that this type of genetic vaccine, encoding multiple viral antigens may be an excellent candidate to include in different vaccine platforms such as mRNA or protein to provide long-term protection against severe disease and potentially against highly mutated future variants of SARS-CoV-2 and related coronaviruses.

## Material and methods

### Study design and participants

We conducted a first-in-human, randomized, placebo-controlled, and double-blind phase 1 clinical trial with a dose escalation design, at the Phase I unit of Karolinska University Hospital, Sweden. The sponsor was Karolinska Institutet, with approval of the study by European Medicines Agency (EudraCT number 2022-001693-63) and the Swedish Ethical Review Authority (Dnr 2022-04103-01).

The key inclusion criteria were age 18–60 years, having received three doses of registered mRNA vaccines with the last dose given ≥3 months prior to inclusion, no clinically significant laboratory abnormalities, negative HIV/hepatitis C virus/hepatitis B virus tests at screening, a BMI of 20–30 kg/m^2^, and willingness to use highly effective methods of contraception. The key exclusion criteria were a history or presence of pulmonary disorders, clinically relevant history of other specified organ system disorders, thrombocytopenia or bleeding disorders, a positive pregnancy test, use of immunosuppressive drugs, vaccination within 2 weeks prior to study vaccine or planned to receive a licensed vaccine before study month 3, and a history of severe adverse reactions to previous vaccines.

A total of 16 healthy volunteers at the ages of 18–60 were enrolled and randomized to receive either the active vaccine or placebo. They received the study drug injections between February and December 2023. All subjects signed an informed consent form prior to enrollment in the study.

### Trial procedures

Three different dose levels of the OC-007 DNA vaccine or of placebo (0.9% NaCl solution) were administered intramuscularly by *in vivo* EP. Six participants in the low-dose group received either 0.5 mg of the study vaccine (*n* = 4) or placebo (*n* = 2). Five participants in the mid-dose group received 1.0 mg of the study vaccine (*n* = 4) or placebo (*n* = 1), and five in the high-dose group received 2.0 mg (*n* = 4) or placebo (*n* = 1). Inclusion was staggered, starting with the lowest dose, with a 1-week interval between each study participant. The Data Safety Monitoring Board (DSMB) reviewed the safety data before permitting escalation to the next dose level.

The EP was performed using the Cliniporator EPS02 pulse generator and the EPS-Gun (IGEA, Italy), a CE-marked electrode dedicated to electro gene transfer vaccination.^11^^,^^12^ The device generated two different intensity pulses. The high 240 V/cm pulse lasted 1 ms, and the low voltage 24 V/cm pulse lasted 400 ms, with a 1-s interval between them. Both pulses resulted in slight involuntary muscle contractions. EP enhances the delivery of DNA into cells and tissues, which is known to improve immune response.^27^

The study participants were assessed at the screening visit, on the day of injection (day 0), and at follow-up visits conducted 1 day (phone contact), 7 (±1) days, 14 (±1) days, 28 (±3) days, and 3 (±14 days) months post-vaccination.

### Safety and tolerability assessments

The primary objective of this trial was to assess the safety and reactogenicity of the OC-007 DNA vaccine delivered by *in vivo* EP. The local (pain at the injection site, redness, and swelling) and systemic reactogenicity (fever, fatigue, headache, chills, vomiting, diarrhea, new or worsened muscle pain, and new or worsened joint pain) were collected for up to 7 days following the vaccine/placebo dose, through a patient diary. The levels of pain, measured by a VAS, were reported immediately (0 min) and at 5, 15, 30, and 60 min post-vaccination. Unsolicited AEs were assessed from the time of the dose application up until 28 days post-vaccination. Samples were collected for hematology, clinical chemistry, and coagulation analyses at screening, on the day of vaccination, at 7 days post-vaccination, and later if deemed necessary. SAEs and suspected unexpected serious adverse reactions were assessed from the study dose until the end of the study, at 3 months post-vaccination.

### Efficacy

Secondary objectives of the trial were antibody (blood and saliva) and T cell (blood) responses. Samplings for immunological analyses of humoral and cellular responses were done on day 0 (prior to vaccination) and at 14 days, 28 days, and 3 months post-vaccination. At each time point, venous blood and saliva samples were collected and later tested for antibody levels against the vaccine components (blood), *in vitro* NT of two SARS-CoV-2 strains (blood), and detection of specific T cells to peptide pools by IFNγ ELISpot assay (blood).

### Detection of IgG-specific antibodies

Serum IgG antibodies against S, RBD, or N were measured by ELISA. In brief, plates were coated with 1 μg/mL of recombinant S, RBD (The Royal Institute of Technology; KTH), or N protein (Genscript) in 50 mM sodium carbonate buffer pH 9.6 overnight at 4°C. Plates were blocked by incubation with dilution buffer (phosphate-buffered saline, 2% goat serum, 1% BSA) for 1 h at 37°C. Serum was added in serial dilutions with a starting dilution of 1:100. Serum antibodies were detected by a peroxidase-conjugated goat anti-human IgG (A0170 Sigma) at 1:30,000 and visualized using TMB substrate solution. Optical density (OD) was read at 450 nm with a 650-nm background. Antibody titers were determined as endpoint serum dilutions at which the OD value was at least three times the OD of the negative control (serum from healthy volunteers prior to the pandemic) at the same dilution.

### Neutralization of SARS-CoV-2 *in vitro*

Titer of neutralizing antibodies (NAbs) in human sera was determined by cytopathogenic effect (CPE)-based microneutralization assay. Briefly, serum was heat inactivated at 56°C for 30 min before a 2-fold serial dilution. Each dilution was conducted in quadruplicate and mixed with 500 plaque-forming units of SARS-CoV-2 huCoV-19/WH1, Beta, Delta, or Omicron in a 1:1 dilution. After 1 h of incubation at 37°C and 5% CO_2_, 100 μL of serum-virus mix was added to Vero E6 cells on a 96-well plate (20 × 10^4^ cells/well) and incubated for 72 h at 37°C and 5% CO_2_. CPE for each well was determined using a Nikon Eclipse TE300 microscope as described.^11^ As controls, wells with medium only, diluted serum only, virus only, and serum known to contain SARS-CoV-2 NAbs mixed with virus were included in each experiment.

### Detection of specific IFNγ-producing T cells by ELISpot

PBMC was collected using SepMate isolation protocol and frozen at −80°C. PBMCs were thereafter tested to evaluate the presence of specific T cells based on IFNγ secretion after peptide or protein stimulation for 36 h using ELISpot Plus: Human IFNγ (ALP) (Mabtech). A total of 100,000 rested PBMCs were cultured in duplicates using different SARS-CoV-2 RBD, M, and N peptide pools. 20-mer peptides with 10 amino acid (aa) overlap, corresponding to the S (127 peptides), RBD (25 peptides), M (22 peptides), and N (41 peptides) and bat-CoV N (42 peptides), were purchased from Sigma-Aldrich (St. Louis, MO). Pools of various peptides were used for the stimulation.

### Activation-induced marker assay

Thawed and rested PBMCs in complete RPMI supplemented with DNAse were stimulated at 1 × 10^6^ cells per well with SARS-CoV-2 peptide pools (0.5 μg/mL each) or an equivalent amount of DMSO as negative control. The following SARS-CoV-2 peptide pools are used: membrane (22 peptides) and nucleocapsid (81 peptides) from Sigma-Aldrich^23^ and spike wild-type and spike Omicron (B.1.1.529/BA.1) peptide pools from JPT. Brefeldin A, monensin, and an anti-CD107a PE-Cy5 (clone eBioH4A3, eBioscience) antibody were added 1 hour later. After 12 h, cells were washed with fluorescence-activated cell sorting buffer (PBS with 2% fetal bovine serum and 2 mM EDTA); stained for viability for 10 min at room temperature, chemokine receptors for 10 min at 37°C, and surface markers for 30 min at room temperature; and then fixed and permeabilized using the FoxP3 Transcription Factor Staining Buffer Set (Thermo Fisher). Intracellular staining (including for activation-induced markers (AIMs) CD40L, CD69, 4-1BB, and IFNγ) was performed for 30 min at room temperature. Finally, cells were washed, resuspended in PBS, and acquired on a Cytek Aurora spectral flow cytometer. Responding CD4+ and CD8+ T cells were defined by expression of AIMs, which are gated as CD69+ CD40L+ CD4+ memory cells and CD107a+ 4-1BB+ and/or IFN-γ+ CD8+ memory cells, respectively. AIM+ frequencies were calculated by subtracting the negative control frequency from the peptide-stimulated frequency. Net frequencies <0.01% or with <5 AIM+ cells were set to 0.01%.

### Statistical analysis

Continuous variables were expressed as mean with ±standard deviation (SD) or median (min-max). Categorical variables were presented as numbers with frequencies, and the proportions were compared using the non-parametric Fisher’s exact test or Mann-Whitney U test. Statistical significance was considered when *p* values were <0.05. Data analyses were performed using GraphPad Prism 10 (10.3.1) for Mac OS.

## Data and code availability

All data are available upon request.

## Acknowledgments

This study was supported by the OPENCORONA consortium, funded by the 10.13039/501100007601European Union’s Horizon 2020 research and innovation program under grant agreement 101003666. The study was also supported by the 10.13039/501100004359Swedish Research Council (M.S. and A.M.), the 10.13039/501100002794Swedish Cancer Society (M.S.), 10.13039/501100004047Karolinska Institutet (M.S.), Region Stockholm, 10.13039/501100018713Center for Innovative Medicine and ALF grants (M.S.), 10.13039/501100004047Karolinska Institutet (L.F.), and by a private donation (M.S.). Parts of this work were carried out within the Karolinska ATMP Center, benefiting from its infrastructure, expertise, and collaborative environment for the development of gene and advanced therapy medicinal products (GTMPs/ATPMs).

## Author contributions

Study design, M.S., G.A., S.Aleman, P.L., P.B., K.T., and A.M.; providing vaccine and delivery device, O.T., E.-K.G., S.S., and M.C.; analysis of human samples, G.A., J.Y., C.B.A.N., A.A., S. Appelberg, and M.B.; producing recombinant proteins, H.T. and S.H.; providing regulatory advise and support, O.L., U.L., K.T., S. Aleman, M.S., M.C., and G.A.; performing clinical trial, S. Aleman, P.L., and P.B.; analyzing data and writing manuscript, M.S., G.A., S. Aleman, P.L., L.F., and A.M.; reviewing and editing manuscript, all coauthors.

## Declaration of interests

M.S. and L.F. are board members and shareholders of SVF Vaccines AB that holds the intellectual property for the vaccine gene used in the clinical trial. G.A. is a consultant to SVF Vaccines AB. M.C. and S.S. are employees of IGEA Medical Spa, Carpi, which owns the EPSgun and Cliniporator used in the clinical trial. M.C. is a shareholder of IGEA. O.T. and E.-K.G. are employees of NorthX Biomedical, Matfors, which produced the DNA vaccine used in the clinical trial. U.H. and O.L. were employed by Scantox, which performed the toxicological evaluation of the study drug for the clinical trial.

## Supplemental information

Document S1. Table S1 and Figures S1–S6

Document S2. Article plus supplemental information
