# Supplementary material for: A phase 1 clinical trial of a multi-antigen SARS-CoV DNA vaccine as a booster after dose three spike-based mRNA vaccinations
Source: Mol Ther Adv. 2026 Feb 28;34(2):201710. doi: 10.1016/j.omta.2026.201710 (PMC13148892; doi:10.1016/j.omta.2026.201710)
Supplement: Document S1. Table S1 and Figures S1–S6 [file mmc1.pdf]

OMTA, Volume *34*

Supplemental information

A phase 1 clinical trial of a multi-antigen SARS-CoV DNA vaccine as a booster after dose three spike-based mRNA vaccinations

Soo Aleman, Gustaf Ahlén, Jingyi Yan, Per Ljungman, Peter Bergman, Christian An Binh Nordentoft, Sofia Appelberg, Arlisa Alisjahbana, Matteo Cadossi, Simona Salati, Katja Tobin, Ola Tuvesson, Hanna Tegel, Eva-Karin Gidlund, Friedemann Weber, Olivia Larsson, Urban Höglund, Marcus Buggert, Sophia Hober, Lars Frelin, Ali Mirazimi, and Matti Sällberg

AE, number÷	0.5 mg dose +EP group (n=4)	1.0 mg dose +EP group (n=4)	2.0 mg dose +EP group (n=4)	All doses+ EP group (n=12)	Possibly or likely related to study procedures	Placebo +EP group (n=4)	Possibly or likely related to study procedures
Pain at the time of vaccination	4	4	4	12	12	4	4
Extended pain at the injection site	0	0	1	1	1	0	0
Tiredness	1	4	4	9	9	1	1
Redness at injection site	2	1	0	3	3	3	3
Local edema at injection site	1	0	0	1	1	1	1
Bruises	0	2	0	2	0	1	1
Headache	1	0	1	2	1	0	0
Diarrhea	1	1	1	3	3	1	0
Thorax pain	0	1	0	1	0	0	0
Hyperesthesia	0	0	0	0	0	1	0
Joint pain	1	0	0	1	0	0	0
Endometriosis	0	0	1	1	0	0	0
SARS-CoV-2 infection	0	0	2	2	0	1	0
Upper respiratory infections	1	2	1	4	0	1	0
Urogenital infections	0	0	1	1	1	1	0
Laboratory abnormalities	3	0	0	3	3	3	0

Table S1. Background data on all healthy volunteers enrolled in the phase I study

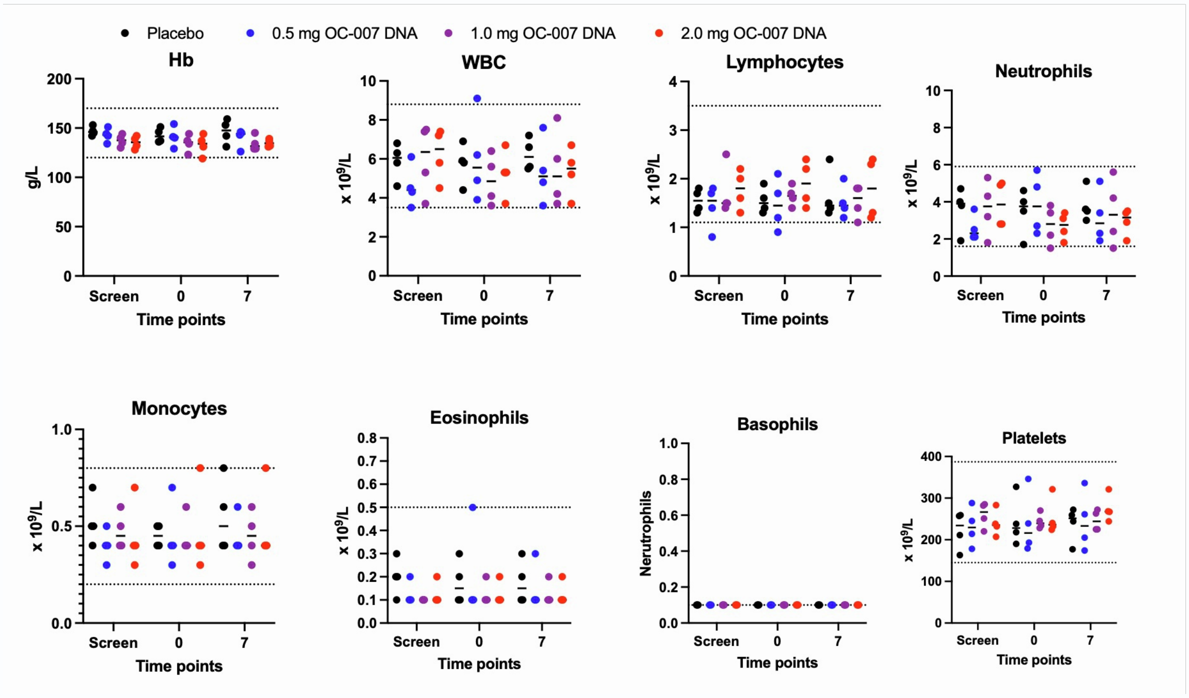

Figure S1. Individual data from the hematological analysis Each dose group is represented by a colour.

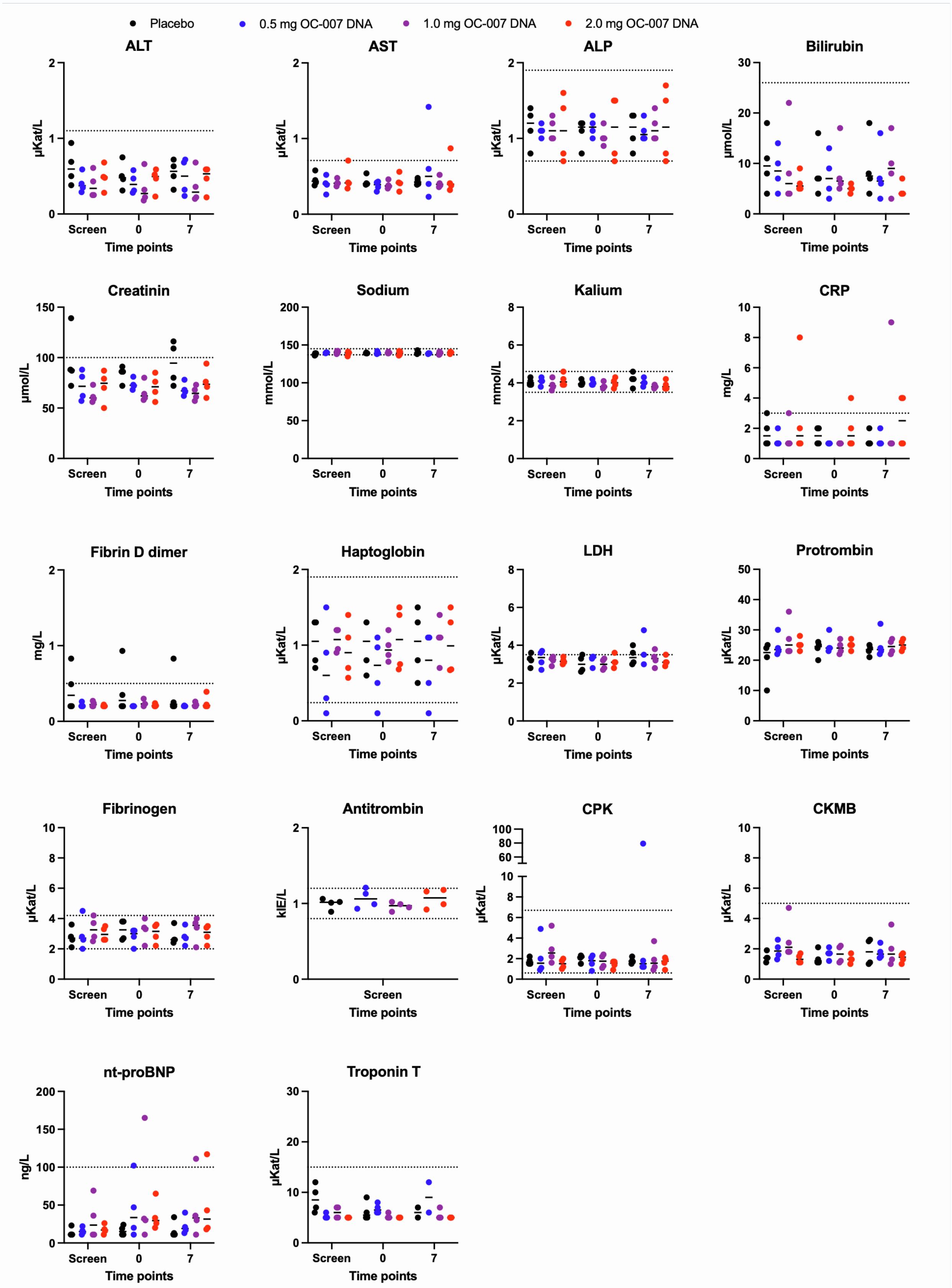

Figure S2. Individual data from the biochemical analysis. Each dose group is represented by a colour.

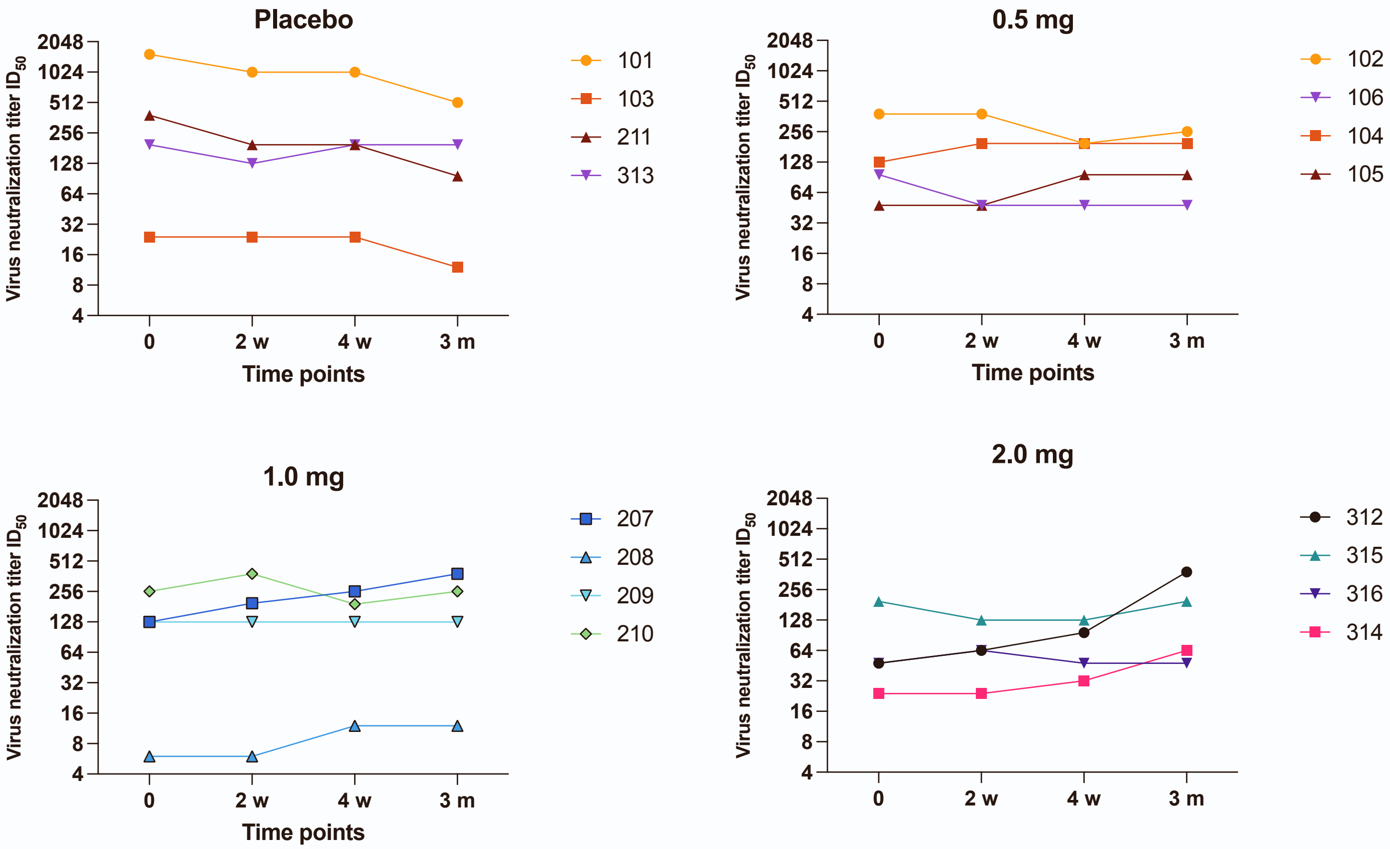

SARS-CoV-2 live virus NT50 (Beta variant)

Placebo group: 0/4 had one or more post vaccination value above base line
OC-007 groups: 8/12 had one or more post vaccination value above base line

p=0.0769, Fisher's exact test

Figure S3. Individual neutralization (NT) end point titers against live SARS-CoV-2 Beta variant virus assay at 50% inhibition. Each subject group is represented by a separate line.

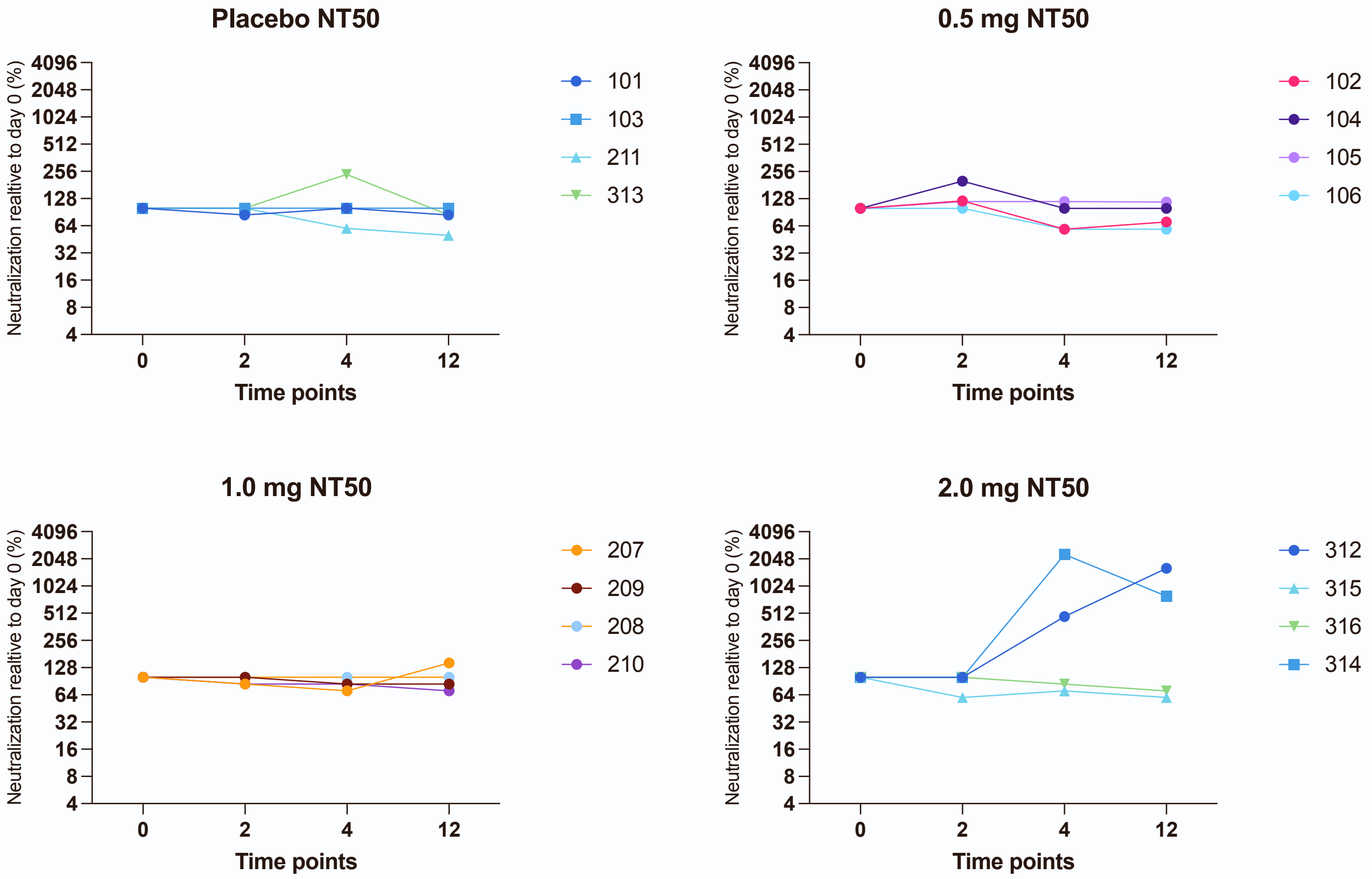

SARS-CoV-2 live virus NT50 (XBB variant)

Placebo group: 1/4 had one or more post vaccination value above base line
OC-007 groups: 5/12 had one or more post vaccination value above base line

p=0.2445, Fisher's exact test

Figure S4. Individual neutralization (NT) end point titers against live SARS-CoV-2 Omicron XBB variant virus assay at 50% inhibition. Each subject group is represented by a separate line.

Activated CD4+ and CD8+ T cells

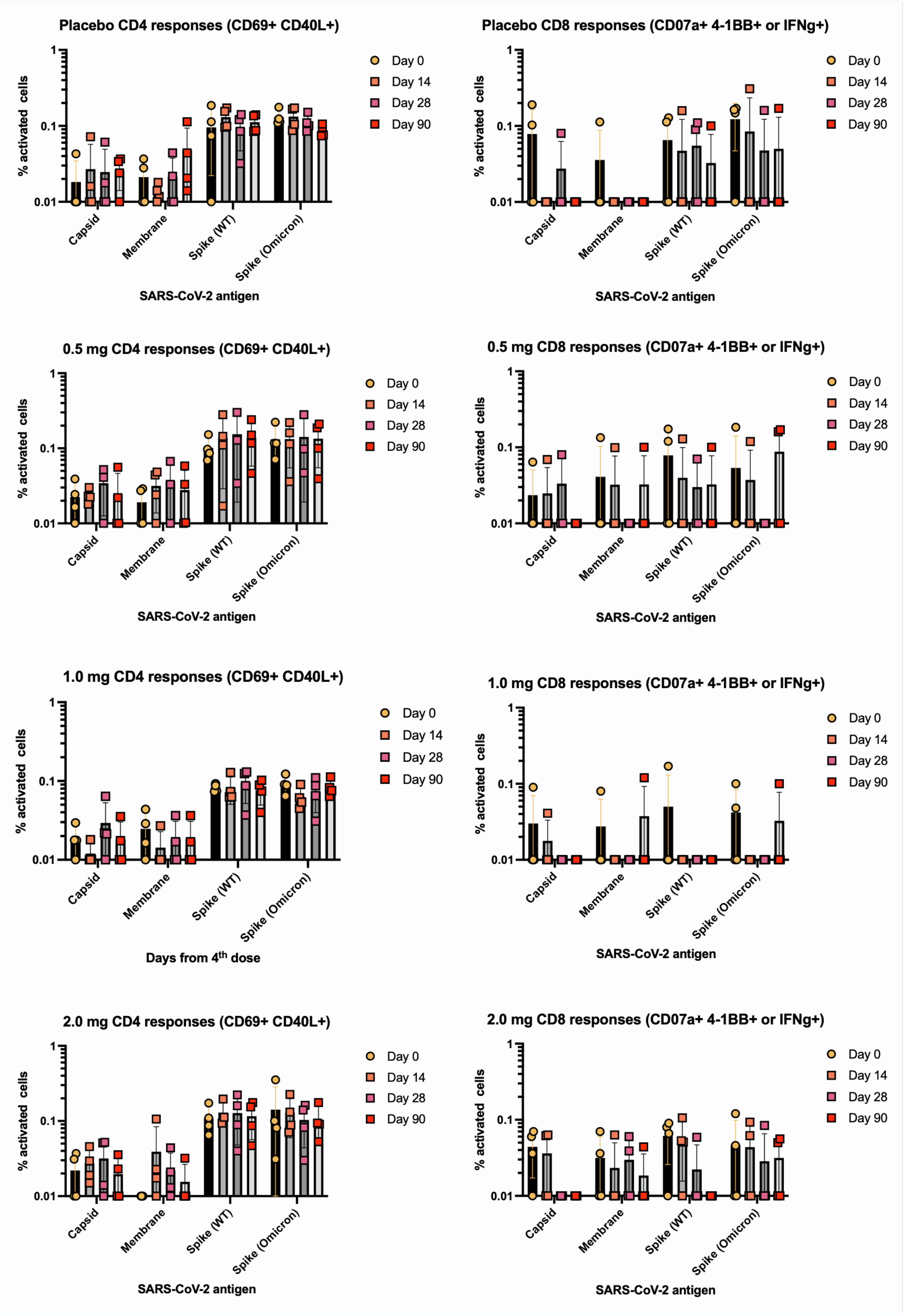

Figure S5. Flow cytometric analysis to peptide pools spanning the nucleocapsid, membrane, and spike (WH-1 and Omicron) proteins of SARS-CoV-2. Data has been given at the mean percent activated CD4+/CD69/CD40L+ and CD8+/CD07a+/4-1BB+ or IFNg+ cells.

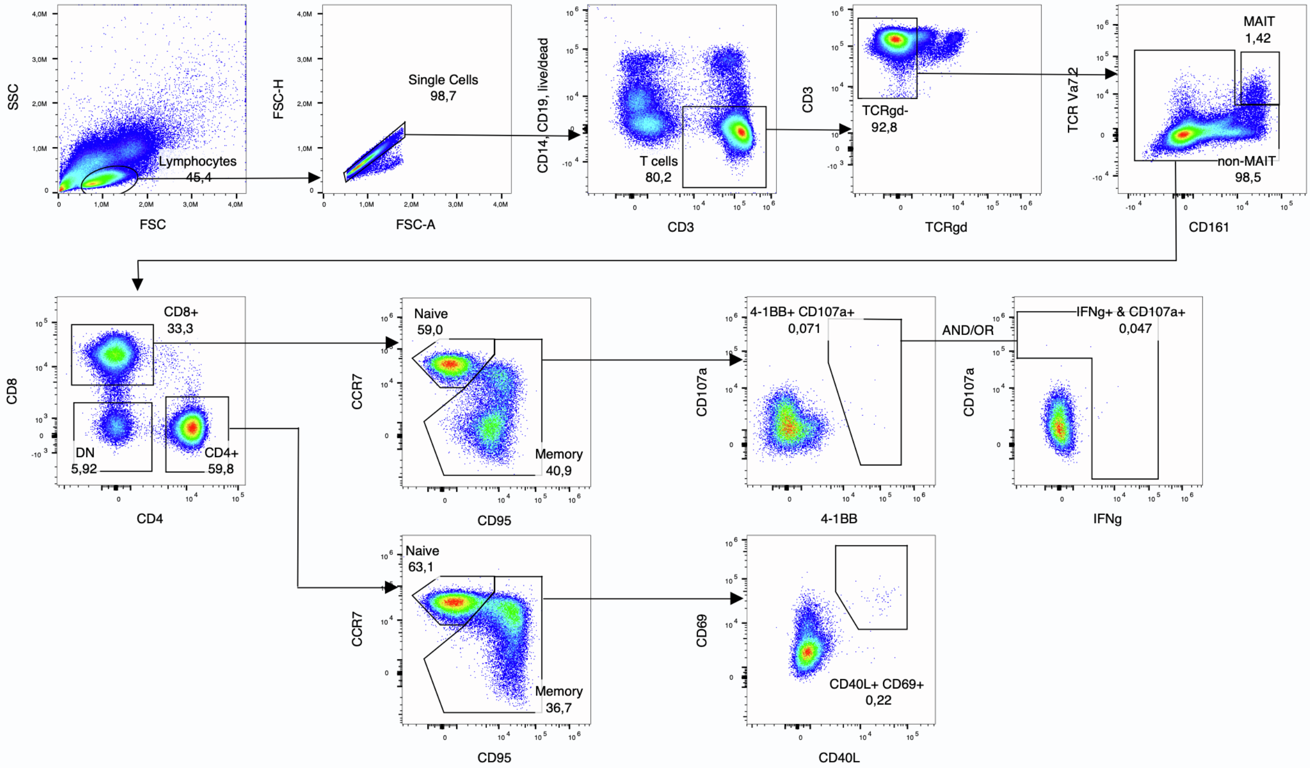

Figure S6. Gating strategy in the AIM assay
